# Food Supply Chain Safety Research Trends From 1997 to 2020: A Bibliometric Analysis

**DOI:** 10.3389/fpubh.2021.742980

**Published:** 2022-02-03

**Authors:** Jianli Luo, Shujuan Leng, Yanhu Bai

**Affiliations:** Department of Finance, School of Business, Wenzhou University, Wenzhou, China

**Keywords:** food supply chain, food safety, bibliometrics, pandemic, traceability

## Abstract

**Background:**

The COVID-19 pandemic has exposed the fragility of the global food supply chain, strengthened consumers' awareness of the traceability system throughout the supply chain, and gradually changed consumers' consumption concepts and consumption patterns. Therefore, the aim of this study was to analyse the relevant literature on food safety in the food supply chain, examine its current status, hot spots, and development trends, and provide some suggestions for academics and relevant government departments in food supply chain safety research.

**Methods:**

We collected the literature on the food safety research of the food supply chain from the Scopus database, used BibExcel to count the subject categories, published journals, geographical distributions, research institutions, authors, and keywords in the literature, and used Pajek software to analyse the keywords in the literature, perform co-occurrence analysis, draw related knowledge maps, and perform cluster analysis on primary keywords. Finally, to study the development trend, we used CorTexT software to illustrate the theme evolution path map in this research field.

**Results:**

The keyword visualization network revealed the following key research topics: (1) food safety at the consumer end of the food supply chain, (2) food safety management in the food supply chain, (3) risk management of food safety in the food safety chain, and (4) food safety at the production end of the food supply chain.

**Conclusions:**

After comprehensive discussion and analysis, we concluded that food supply chain management may be a hot topic in the future, especially in traceability management combined with the blockchain. It is necessary to explore in-depth how the blockchain can affect the food supply chain to provide a theoretical basis for managing the latter.

## Introduction

The COVID-19 pandemic that started in 2020 threatens global food safety in the food supply chain; the main concern revolves around the sources of food safety risks, which alerted stakeholders to the need to revise food safety risk management strategies globally (1). The food industry is becoming increasingly aware of the fragility of agricultural products, the uncertainty of the food supply, and the flexibility of transportation and logistics (2), which have attracted increasing attention from scholars aiming to study the close relationship of food safety with the food supply chain.

**Figure 1:**
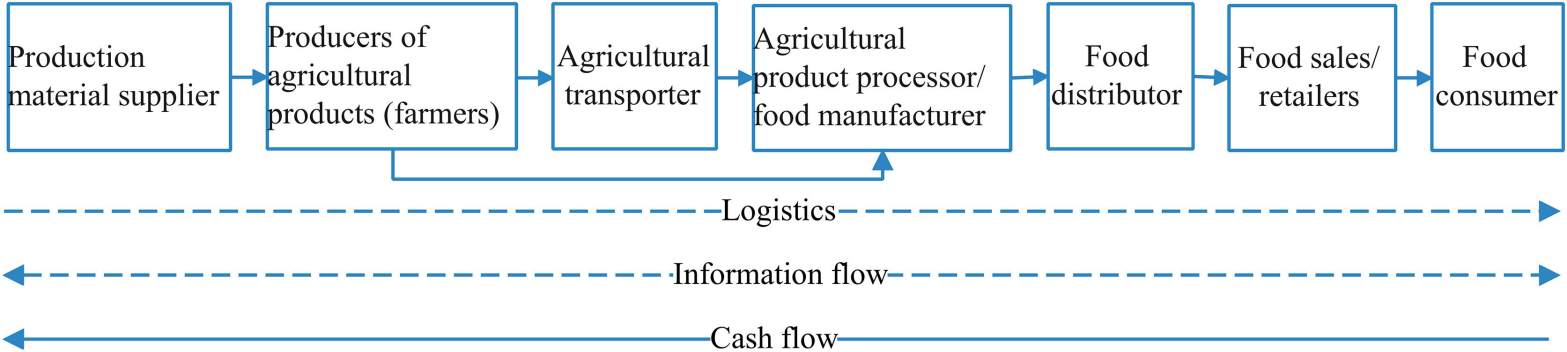
The structure model of the food supply chain.

Den Ouden et al. (3), scholars of agriculture and biology, first proposed the food supply chain, which is a network structure consisting of consumers of agricultural products, food production, the processing, food logistics, and distribution industries, food sales companies, and related entities (4, 5). A simplified food supply chain structure model is shown in Figure 1 (6). Food has unique attributes such as corrosion and environmental impact (7), combining the food supply chain's complex characteristics, networked organizational structure, and dynamic supply network (8). Food contamination is a significant food safety risk in all aspects of the food supply chain, such as production, procurement, processing, circulation, and sales (9, 10). Therefore, this article discussed the food supply chain safety from the aspects of food quality, health, and biosafety.

As people pay increasing attention to food safety, researchers are beginning to review the relevant articles in the field. Auler et al. (11) systematically reviewed 46 articles on food safety in the field of supply chain management and revealed the main features of the literature in this research field. Wahyuni et al. (12) reviewed the titles and abstracts of articles on food safety and halal food in the supply chain and made a cluster analysis of the research network in this field but did not discuss related topics in depth. Azmi et al. (13) studied the types of risks involved in the halal food supply chain, thereby offering important insights into the strategic development and integrity of the halal supply chain. However, these studies focused on performing traditional literature reviews and only studied some aspects of the food supply chain, thereby failing to comprehensively outline the development of food supply chain safety.

In this study, we used existing literature reviews with bibliometrics analysis methods, analyzed quantitatively the development status and research hotspots of food supply chain safety, and predicted its future development trend. The specific objectives of this study were: (1) to determine the prominent research profile in the food supply chain safety research field, such as research disciplines, influential journals, and geographical distribution; (2) to determine the key themes of analysis in this research field; and (3) to determine the evolution path and future development trend of this research field.

The main contributions of this study are the following: (1) we analyzed comprehensively the research status and disciplinary characteristics of the food supply chain safety field; (2) we discussed comprehensively the evolutionary path of this research field; (3) we pointed out future research priorities for scholars, such as consumer trust in the food supply chain, food supply chain traceability, blockchain application, and risk management.

**Figure 2:**
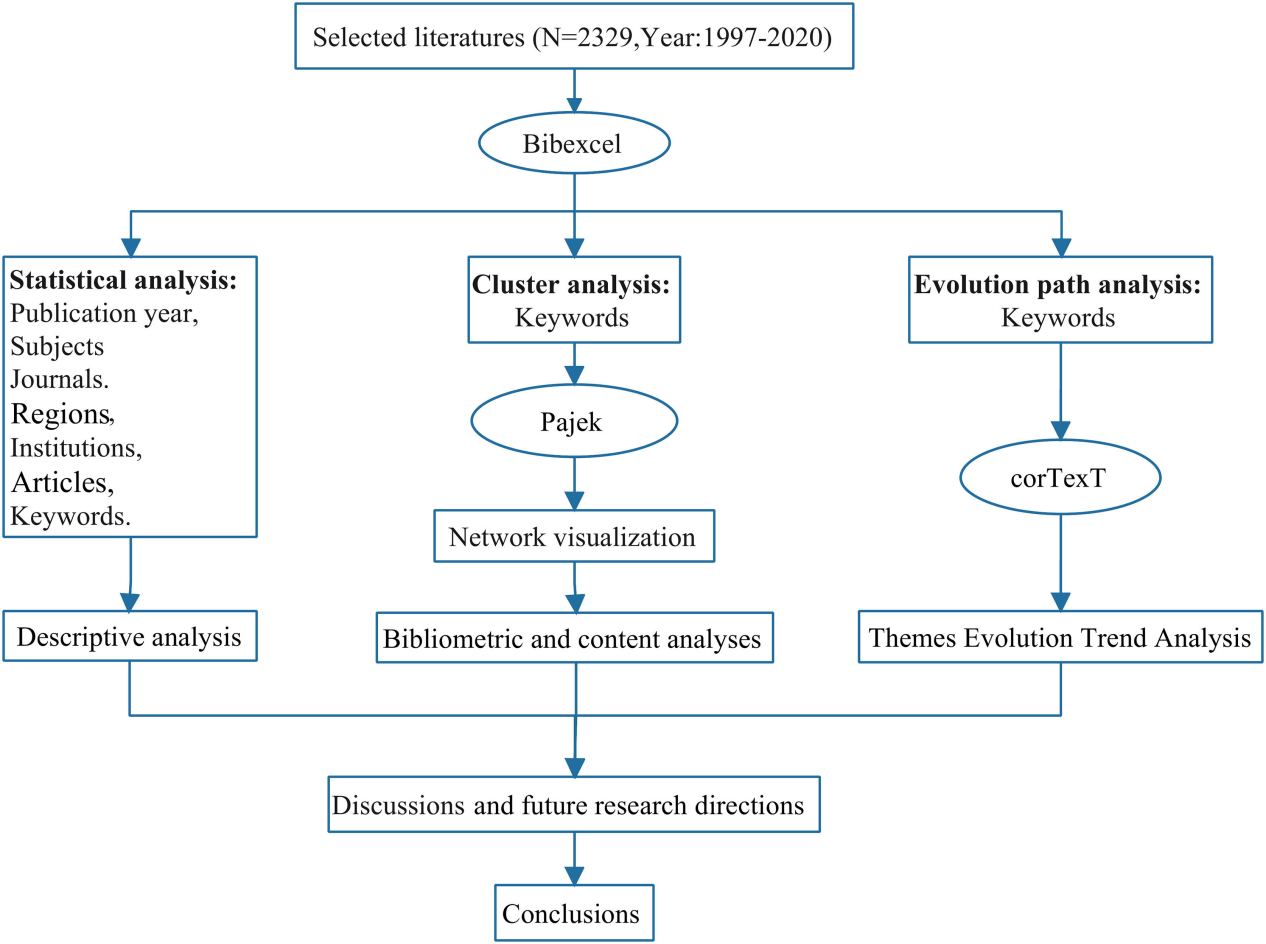
Article structure diagram.

The rest of this article is organized as follows (refer to Figure 2): in Section Materials and methods, we discuss research methods and the initial statistical analysis of the data; in Section Results, we present our research results and analysis, descriptive statistics, cluster analysis, and evolutionary path analysis of the selected literature; in Section Discussion, we discuss the findings of this research and propose future research directions; in Section Conclusions, we summarize our main conclusions.

## Materials and Methods

### Bibliometrics Analysis

In this study, we used bibliometrics to comprehensively analyse the food safety-related literature in the food supply chain and dig deeper into the quantitative and qualitative characteristics of the literature to help researchers to evaluate the food safety field in the food supply chain and the research path and research trend of the research as well as assist scholars in implementing innovative ideas based on existing literature.

Bibliometric analysis is a cross-science analysis that integrates mathematics, statistics, and philology and employs mathematical and statistical methods to quantitatively analyse the data from all research databases (14). Through citation analysis, co-citation analysis, statistical analysis of the title, author, journal, country, institution, reference, and subject category in the bibliographic information of a particular field, bibliometrics can evaluate the development trend of the literature, the research subject, and prominent research institutions, periodicals, essential documents, influential citations, and other comprehensive document systems (15). The main aim of the bibliometric analysis is to analyse keywords and use the co-occurrence of vocabulary pairs or noun phrases in the literature to determine the relationship between the topics (16).

### Thematic Evolution Trend Analysis

Thematic evolution analysis is a new research method developed recently in the information science field that is widely used in many disciplines. It can better identify the subject development, evolution, and flow of a particular research field in a certain period, thereby assisting researchers in understanding more comprehensively the development of a specific field. In this study, we used CorTexT to draw the evolutionary path diagram; the length of the topic direction on the ordinate axis indicates the proportion of the total frequency of keywords in that direction. The expansion and contraction of the alluvial area represent the scale change in different time intervals.

### Analysis Tools

In this study, we used the document processing tool BibExcel and network analysis tools Pajek and CorTexT Platform (www.cortext.net). Bibexcel performs basic statistical analysis on the number of articles, citations, and *h*-index of authors, journals, and countries in the bibliographic information downloaded from the Scopus database (17). The visualization software Pajek performs bibliographic analysis, citation analysis, co-citation analysis, and cluster analysis of related data (18). Finally, CorTexT reveals the evolutionary characteristics of food safety research topics in the food supply chain over time (19).

### Data Sources and Processing

In this study, we obtained research data from Scopus, the largest abstract and citation database of peer-reviewed literature and international publishers globally that provides a one-stop platform for scientific researchers to access the scientific literature. We employed four steps while using Scopus: keyword identification, selection criteria for inclusion and exclusion, quality evaluation, and data extraction (20).

The term “food safety in the food supply chain” comprises three key elements: food, supply chain, and safety; therefore, we included three search strings to ensure that relevant literature data were obtained. The first search string contained keywords related to food according to the agricultural commodity keywords defined by the Food and Agriculture Organization of the United Nations: food^*^ or dairy or fruit or grain or cereal or meat or pork or beef or chicken or fish or vegetable or grape or wine or rice or coffee or oil or horticulture or “sugar cane” or maize or wheat or potato or “sugar beet” or soybeans or cassava or tomato or barley or cotton or apple. The second string consisted of keywords of supply chain-related terms: “supply chain” or “supply network” or “demand chain” or “value chain” or purchas^*^ or sourc^*^ or logistics or procurement. The third-string consisted of security-related keywords: safet^*^ or securit^*^ or risk^*^ (refer to Table 1).

**Table 1:** Topic search queries used for data collection.

**Set Records Search Queries**		
#1	3,424,449	(TITLE(food* OR dairy OR fruit OR grain OR cereal OR meat OR pork OR beef or chicken OR fish OR vegetable OR grape OR wine OR rice OR coffee OR oil OR horticulture OR “Sugar cane” OR maize OR wheat OR potato OR “sugar beet” OR soybeans OR cassava OR tomato OR barley OR cotton OR apple)) OR (KEY(food* OR dairy OR fruit OR grain OR cereal OR meat OR pork OR beef or chicken OR fish OR vegetable OR grape OR wine OR rice OR coffee OR oil OR horticulture OR “Sugar cane” OR maize OR wheat OR potato OR “sugar beet” OR soybeans OR cassava OR tomato OR barley OR cotton OR apple))
#2	1,312,930	(TITLE(“supply chain” OR “supply network” OR “demand chain” OR “value chain” OR purchas* OR sourc* OR logistics OR procurement)) OR (KEY(“supply chain” OR “supply network” OR “demand chain” OR “value chain” OR purchas* OR sourc* OR logistics OR procurement))
#3	4,151,056	(TITLE(safet* OR securit* OR risk*)) OR (KEY(safet* OR securit* OR risk*))
#4	11235	#1 AND #2 AND #3

We searched the “title” and “keyword” fields in the Scopus database, with no time limit for the search, and the resulting records were 11,235 (by 12/31/2020). We found the literature retrieved from the Scopus database was first published in 1997. In addition, the Codex Alimentarius Commission issued the “Hazard Analysis and Critical Control Point (HACCP) System and Guidelines for its Application” for food safety and hygiene in 1997, thus providing outline requirements for global food safety management and certification. In the same year, the United States allocated an additional $100 million to launch a food safety program, the European Union began phasing in a traceability system for food information, and Britain's Department for Environment, Food, and Rural Affairs set up a livestock traceability system. So, we considered 1997 as the starting point of the research topic.

**Figure 3:**
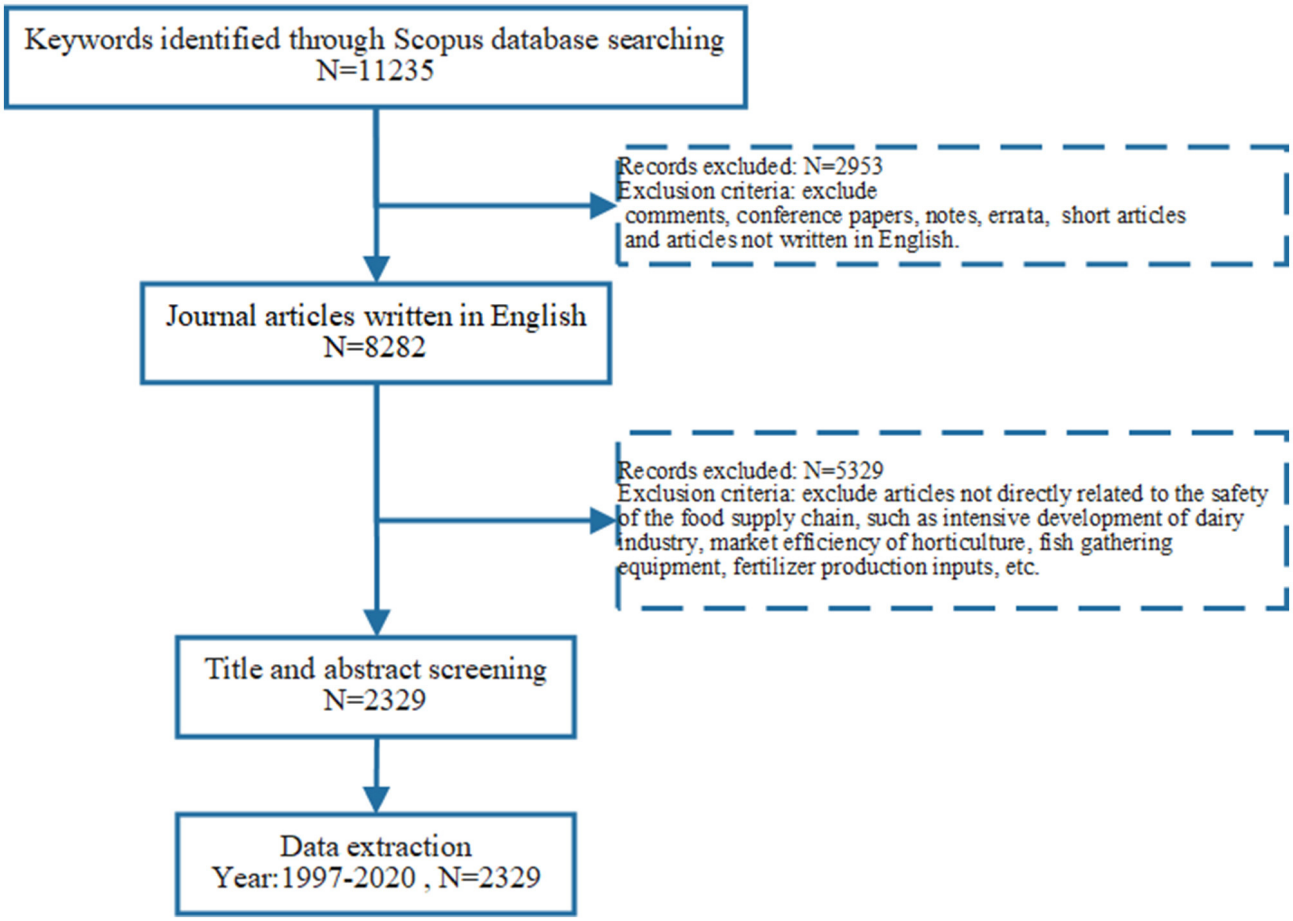
Literature selection process.

The search scope was limited to “journal articles” written in “English”, while comments, conference papers, notes, errata, and short articles and surveys were excluded. This reduced the resulting records to 8,282. Then, we screened the titles and abstracts of 2,329 articles based on the inclusion and exclusion criteria. Specifically, in this study, we selected articles published in peer-reviewed English-language journals, such as articles discussing various aspects of food safety in the food supply chain (e.g., definitions, agriculture, chemistry, nutrition, biology, food engineering, and quality risk evaluation). We excluded articles that were not directly related to the food supply chain safety, such as those discussing the intensive development of the dairy industry, gardening market efficiency, fish gathering equipment, and fertilizer production input. Finally, 2,329 relevant articles from 1997 to 2020 were selected for bibliometric analysis (as shown in Figure 3).

The aforementioned processes were performed by two collaborators independently screening articles, comparing search results, and reaching an agreement on such as the aforementioned 2,329 articles. The inter-rater reliability was 100%, which ensures the accuracy and rationality of the data analyzed.

## Results

### Descriptive Analysis

**Figure 4:**
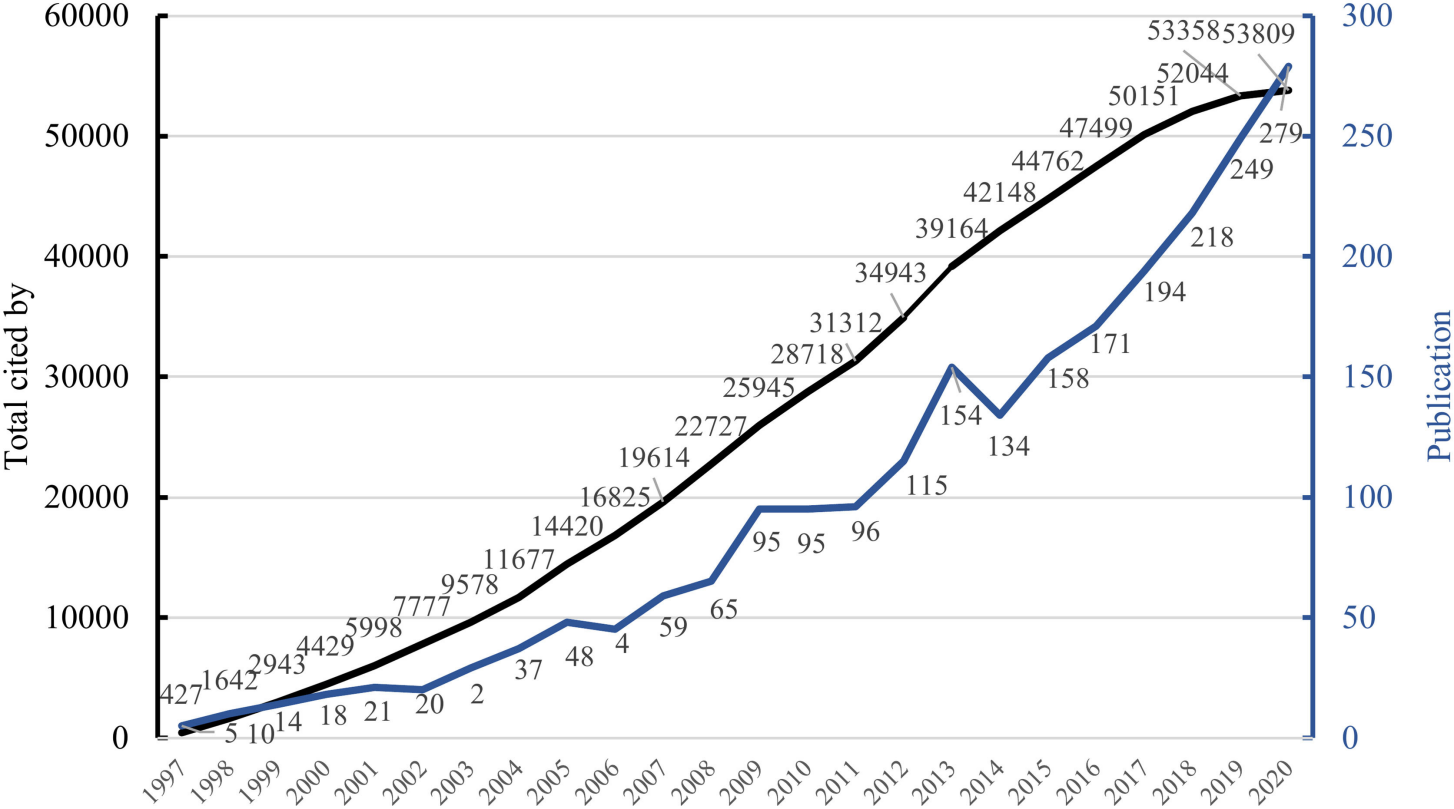
The number of annual publications and total cited between 1997 and 2020.

Figure 4 shows the change in the number of publications on food safety in the food supply chain between 1997 and 2020. It can be seen from statistics that although the number of articles published was at a low period in 2014, it has shown an overall upwards trend. The total number of articles published on food safety in the food supply chain in 2020 is the largest, reaching 279 items, accounting for ~11.98%. Second, the total number of articles in 2019 followed closely, with 249 pieces, accounting for about 10.69%. The number of journal publications on relevant topics peaked in 2015–2020. Figure 4 also shows that the number of times published articles are cited increases continually, which shows that researchers focus on the in-depth and innovative research content of scholars to advance the systematicity of food safety research in the food supply chain. It can be predicted from the current trend that research related to food safety in the food supply chain will continue to grow and the level of study and research content will continue to improve.

#### Subject Categories

**Figure 5:**
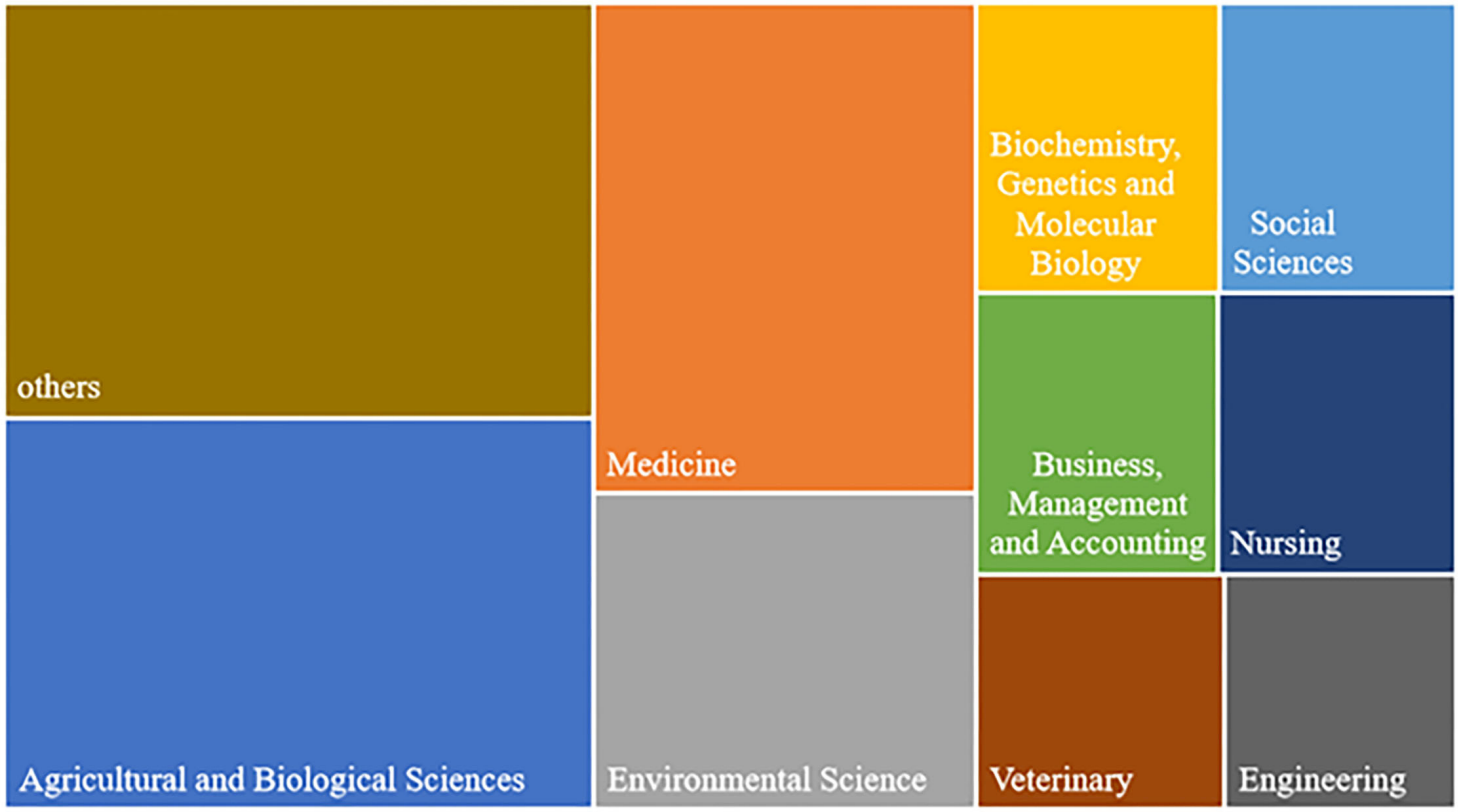
Subject categories.

The 2,329 articles on food safety in the food supply chain are included in our analysis, contained 27 research topic categories according to the Scopus classification. We classify the subject of a single article by the subject category of the source journal. Due to the intersecting nature of food supply chain safety research fields, one article may cover multiple subject categories. We extracted 4,409 subject data from 2,329 pieces of literature by Bibexcel software, involving a total of 29 subject categories. According to different research directions, the main research fields are nine: agricultural and biological sciences, medicine, environmental science, biochemistry, genetics, and molecular biology, social sciences, business, management and accounting, nursing, veterinary, and engineering (refer to Figure 5). As it can be concluded from Figure 5, the most researched direction today is the agricultural and biological sciences one, with 870 articles accounting for ~19.73%. With the advancement of science and technology and the increasing number of food contamination incidents in the food supply chain, practitioners, and scholars have been alerted to food safety issues and their consequences, such as genetic modification technology, genetic modification supervision, and the application of food pesticides.

#### Influential Journals

The total number of journals related to food safety in the food supply chain that published relevant articles from 1997 to 2020 was 182. This demonstrates the extent and variety of publications and discussions in this field. Table 2 lists the top 10 journals in terms of publication volume. Impact factor (IF) is an internationally recognized journal evaluation index. It is generally believed that journals with an IF >1 can be considered valuable journals in social sciences. All the top 10 journals shown in Table 2 had an IF >1, which ranged from 2.304 to 6.766. Additionally, they focused on the impact of food safety issues on the environment and health in the food supply chain as well as risk identification and risk management. The *h*-index measures the citation impact and productivity of publications and aims to quantify the results of researchers as independent individuals. From 1997 to 2020, “Preventive Veterinary Medicine” published the most articles cited the most times, was cited 2,253 times, and had the highest *h*-index (30). Interestingly, among all journals, the IF (6.766) of the “American Journal of Clinical Nutrition” was the highest. Still, its total publications and *h*-index were much lower than those of “Preventive Veterinary Medicine”, which shows that its primary influence was not on the food safety research in the food supply chain.

**Table 2:** The performance of the 10 most leading journals.

**Rank**	**Journal**	**IF_**2019**_**	**CS**	**SJR**	**SNIP**	**TP**	**TC**	**H-Index**
1	Preventive veterinary medicine	2.304	4.1	0.969	1.243	70	2,253	30
2	Food control	4.258	8.4	1.43	1.733	40	1,176	19
3	Science of the total environment	6.551	8.6	1.661	1.977	34	1,653	19
4	Cancer epidemiology biomarkers and prevention	4.344	8.2	2.857	1.729	18	1,199	17
5	Cancer causes and control	2.375	4.2	1.332	0.965	26	1,270	16
6	American journal of clinical nutrition	6.766	12.1	2.704	2.339	18	1,856	16
7	British journal of nutrition	3.334	6.4	1.236	1.297	20	605	14
8	Journal of nutrition	4.281	8.2	1.797	1.644	15	902	14
9	Public health nutrition	3.182	4.8	1.21	1.269	29	733	14
10	Risk analysis	3.137	5.1	1.092	1.482	27	877	14

#### Geographical Distributions

**Figure 6:**
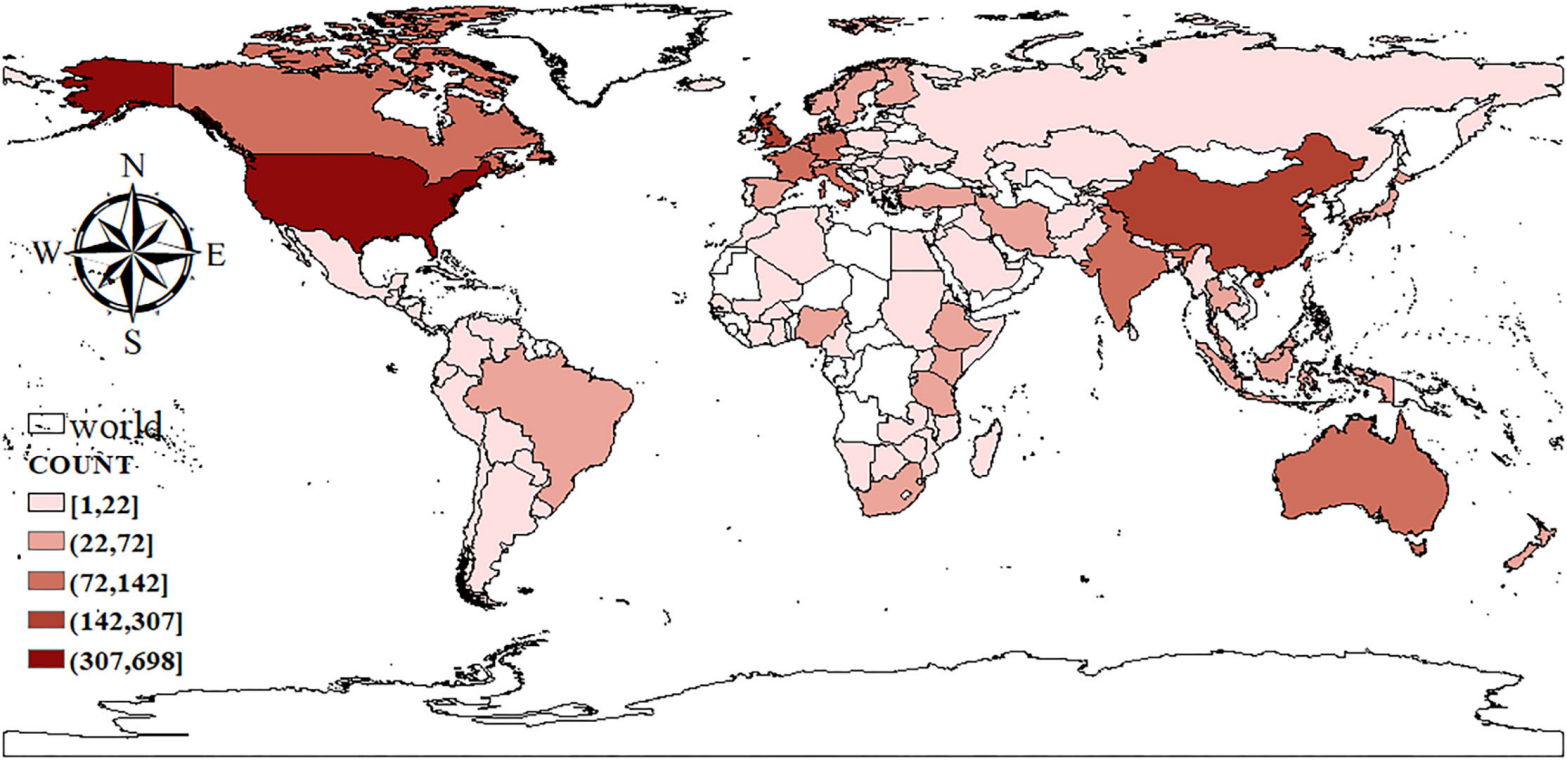
Distribution of national publications.

From 1997 to 2020, researchers from 132 regions published articles on food safety in the food supply chain. The range of areas covered was wide and concentrated in the United States, China, and the United Kingdom. The publication volume in 79 areas was <10, accounting for ~59.85%. In 19 countries/regions, the publication volume was 10–20, accounting for 14.39% of the total; while in 23 countries/regions, the publication volume was 20–80, accounting for ~17.42% of the total (refer to Figure 6). As shown in Table 3, the publication volume in 10 countries/regions is more than 80 articles. The United States, China, and the United Kingdom rank in the top three in terms of publication volume and *h*-index; however, the number of citations of the second-ranked Chinese article is lower than the number of sources of the third-ranked 31 article. Additionally, the *h*-index of China (38) is lower than that of the UK (44), thereby indicating that the articles published by the latter are more influential.

**Table 3:** The top 10 productive countries.

**Rank**	**Country**	**TP**	**%TP**	**TC**	**%TC**	**H-index**
1	United States	698	20.42%	22,853	28.01%	69
2	China	307	8.98%	5,059	6.20%	38
3	United kingdom	212	6.20%	5,695	6.98%	44
4	Canada	142	4.15%	3,931	4.82%	33
5	Australia	134	3.92%	2,607	3.20%	29
6	Italy	123	3.60%	2,536	3.11%	29
7	Netherlands	114	3.33%	4,106	5.03%	37
8	Germany	112	3.28%	2,742	3.36%	28
9	India	90	2.63%	1374	1.68%	20
10	France	83	2.43%	2,115	2.59%	28

%TP = (Total Publications/Document numbers)*100%; %TC = (TC = Total Citations/All Citations)*100%.

#### Influential Institutions

The first authors of the 2,329 articles included in this study represented 167 different research institutions, approximating to the total number of journals (182) mentioned in Influential journals, indicating the breadth of research in food safety in the food supply chain and its interdisciplinary nature. Table 4 lists the 10 most influential institutions that promote the development of this field, of which seven are universities. These institutions are either comprehensive, agricultural, or medical research institutions. They are located in six countries, four of which are the countries that have the highest publication volume globally. Among the research institutions with the most significant number of publications is Wageningen University & Research in the Netherlands, with 68 publications and 2,778 citations.

**Table 4:** The top 10 influential institutions.

**Rank**	**Institution**	**Country**	**TP**	**TC**	**H-index**
1	Wageningen University & Research	Netherland	68	2,778	30
2	Chinese Academy of Sciences	China	59	4,350	29
3	Harvard University	United States	43	2,161	27
4	Swedish University of Agricultural Sciences	Sweden	37	1,365	20
5	University of Milan	Italy	23	519	12
6	University of Copenhagen	Denmark	23	459	10
7	National Cancer Institute (NCI)	United States	21	1,071	16
8	Harvard Medical School	United States	20	959	10
9	Chinese Academy of Agricultural Sciences	China	20	736	9
10	Cornell University	United States	20	200	8

#### Influential Authors

Table 5 lists the 10 most influential authors and their countries of origin, home institutions, publication volumes, citation volumes, and *h*-index. The top author La Vecchia is employed by the University of Milan in Italy and has the highest number of publications (16), citations (602), and *h*-index (12) in this field. This result demonstrates that La Vecchia is among the most influential authors in the food supply chain research field.

**Table 5:** Information on the ten most influential researchers.

**Rank**	**Author**	**Country**	**Institution**	**TP**	**TC**	**H-index**
1	La Vecchia, C.	Italy	University of Milan	16	602	12
2	Talamini, R.	Italy	Centro di Riferimento Oncologico	10	556	10
3	Jacxsens, L.	Belgium	Ghent University	10	340	10
4	Willett, W.C.	United States	Harvard School of Public Health	9	1,715	9
5	Franceschi, S.	France	International Agency for Research on Cancer	9	542	9
6	Mirmiran, P.	Iran	Shahid Beheshti University of Medical Sciences	9	441	8
7	Azizi, F.	Iran	Shahid Beheshti University of Medical Sciences	9	441	8
8	Uyttendaele, M.	Belgium	Ghent University	9	320	8
9	Negri, E.	Italy	University of Milan	9	417	7
10	Grace, D.	Kenya	International Livestock Research Institute	9	103	7

Further analysis of the number of authors in published articles, showed that the average number of authors per publication is 5.47. Among the publications examined, 696 had five authors, 483 had six authors, and 422 had four authors accounting for ~33.46, 23.22, and 20.29%, respectively, of the total number of articles. Surprisingly, articles written by eight or more authors and those written by 10 or more authors accounted for ~9.8 and 3.46%, respectively, of the total. These results suggest that food safety research in the food supply chain involves a considerable workload and requires experiments, surveys, and data collection often requiring the contribution of a team.

#### Frequently Cited Articles

In this study, we used the number of times a publication has been cited to evaluate its performance and scientific excellence; the higher the citation frequency, the greater the influence of the publication. In particular, to more accurately describe the impact of the article, this article excludes the number of self-citations. Table 6 presents the information of the top 10 most cited articles in food safety research articles in the food supply chain from 1997 to 2020. The most cited article and the sixth most cited article were published in 2002 at the “Journal of the National Cancer Institute”; still, in both articles, long-term research data were used to illustrate the products of the production and consumption ends of the food supply chain and what consumers buy and eat (21, 25). Research has shown that the frequent intake of tomato products or lycopene (lycopene carotenoids) can reduce the risk of prostate cancer (21); high-fat dairy products, mostly skimmed/low-fat milk, can reduce the risk of breast cancer (26). This shows that food supply chain scholars attach great importance to the issues closely related to food safety and human health, especially the impact of food in the food supply chain on certain cancers that cannot yet be treated.

**Table 6:** The top 10 frequently cited articles.

**Rank**	**Author/Year**	**Journal**	**TC**	**PC**
1	Giovannucci et al., 2002 (21)	Journal of the National Cancer Institute	570	31.67
2	Kummu et al., 2012 (22)	Science of the Total Environment	459	57.38
3	Hu et al., 1999 (23)	American Journal of Clinical Nutrition	428	20.38
4	Malm, 1998 (24)	Environmental Research	367	16.68
5	Opsomer et al., 2000 (25)	Theriogenology	338	16.90
6	Shin et al., 2002 (26)	Journal of the National Cancer Institute	301	16.72
7	Cornelis et al., 2006 (27)	Journal of the American Medical Association	296	21.14
8	Roth et al., 2008 (28)	Journal of Supply Chain management	282	23.50
9	Ko et al., 1997 (29)	International Journal of Epidemiology	261	11.35
10	Azadbakht et al., 2005 (30)	American Journal of Clinical Nutrition	247	16.47

#### Frequently Used Keywords

In this study, we used the frequency of keywords as a metric to identify sub-areas and topics that have attracted the attention of researchers long-term. Taking into account that in some articles, there were keyword labeling irregularities and the possible lack of keyword fields; in this study, we extracted keywords, removed duplicates to obtain the original record of the keywords without repetition, merged singular and plural forms, abbreviations, and synonyms, and classified keywords into categories. Table 7 shows some of the most frequently occurring keywords. The top 10 keywords are human (822), country (792), statistical model (708), gender difference (640), age distribution (563), controlled study (481), risk factor (474), risk assessment (452), logistic models (451), and foodborne diseases (429).

**Table 7:** Frequently occurring keywords.

**Rank**	**Keywords**	**NO**	**Rank**	**Keywords**	**NO**	**Rank**	**Keywords**	**NO**
1	Human	822	16	Food supply	329	31	Meat	178
2	Country	792	17	Dietary intake	320	32	Vegetable	175
3	Statistical model	708	18	Food supply chain	288	33	Crop production	165
4	Gender difference	640	19	Major clinical study	263	34	Nutritional assessment	164
5	Age distribution	563	20	Health risk	247	35	Food microbiology	154
6	Controlled study	481	21	Dairy product	222	36	Organic pollutants	151
7	Risk factor	474	22	Questionnaire	213	37	Traceability	149
8	Risk assessment	452	23	Income	197	38	Pevalence	148
9	Logistic models	451	24	Environmental impact	197	39	Food contamination	147
10	Foodborne diseases	429	25	Food consumption	196	40	Heavy metal	147
11	Food security	409	26	Agriculture	194	41	Socioeconomics	144
12	Food safety	408	27	Disease association	189	42	Catering service	143
13	Risk	396	28	Consumer behavior	185	43	Adverse effects	140
14	Animal	391	29	Cross-sectional studies	180	44	Supply chain management	134
15	Diet	355	30	Education	180	45	Chemical contamination	134

*NO, Number of occurrences*.

### Bibliometric Analyses

**Figure 7:**
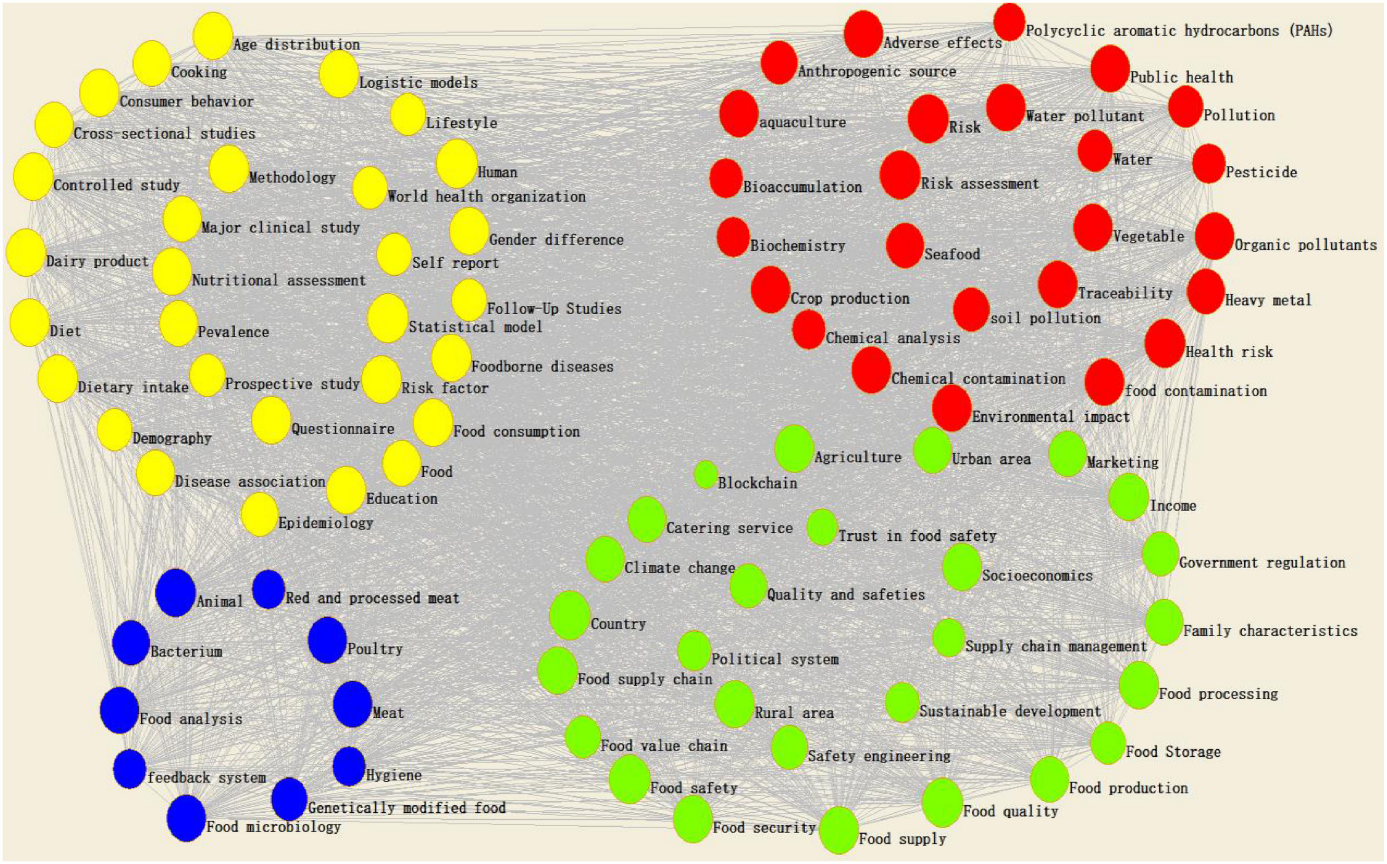
Visualization of keywords' networks (NO > 20).

We selected keywords that appeared more than 20 times for visual analysis and clustering, analyzed and summarized closely related keywords in the visual network, and further analyzed the food safety research in the food supply chain subtopic. Judging from the results of the community division of the topic association network, the current international infographics have formed four research directions (or topic communities) of different scales in significant data research, namely: C1, food safety at the consumer end of the food supply chain; C2, food safety management in the food supply chain; C3, risk management of food safety in the food supply chain; C4, food safety at the production end of the food supply chain (Figure 7). We performed a content analysis based on the four groups to determine the detailed subtopics and insights.

#### Cluster 1 (Yellow): Food Safety at the Consumer End of the Food Supply Chain

In the food supply chain, consumer food safety issues are closely related to consumers, especially consumers' trust in food, safe consumption awareness, and consumer attitudes. Consumers' convictions stem from the food safety information provided by the Environmental Hygiene Department and the Food Standards Agency, especially the food safety information in food packaging (31). However, some food labels lack the information required by consumers, such as nutritional content, production system, traceability, and quality control information. The opacity of food information has led to an increased incidence of foodborne diseases. Gradually, some consumers change their food consumption habits and turn to organic food and food produced with improved safety (32).

Ethical and safe food consumption is not a widely recognized issue and many consumers have not yet developed food safety awareness (33). All localities need to educate consumers on food safety, improve consumers' food safety issues, increase their willingness to buy safe food, and improve local food safety levels (34). The long-term nature of the COVID-19 pandemic also requires consumers to increase their awareness of cold food chain safety, strengthen the maintenance, and understanding of the cold chain within the food framework, assume the responsibility for maintaining the cold chain and reduce the unknown risk of improper food handling (35).

Many factors affect consumers' food safety behaviors in the food supply chain, such as consumers' age, gender, education, income level (36). The rapid development of e-commerce in the information age has strengthened consumers' willingness to buy food online, increased consumer trust, and increased online purchase rates (37). Consumers are gradually choosing a healthy lifestyle and are more inclined to buy organic food, especially millennials, who are willing to buy organic food at a higher price (38). This creates more opportunities for the food industry and has also attracted the academic community's attention, especially in terms of consumer attitudes toward accepting or resisting organic food (39).

#### Cluster 2 (Green): Food Safety Management in the Food Supply Chain

The food supply chain needs to be committed to coordinated management among supply chain members, reduce the mileage of the food supply chain, increase the smoothness of information circulation and food safety, and improve the supply chain's sustainability and globalization. This will enhance the market positioning of all links in the food supply chain, help to launch new products and maintain a high degree of safety and traceability (40). Governments' food and agricultural sector policies should be more comprehensive, make full use of logistics network technology, establish a safety information platform, and monitor food safety issues in real-time (41). Countries need to develop food safety agencies, service supply chain management, establish sustainable food supply and food distribution logistics models, and prove the affordability and sustainability of the food supply chain (42). In particular, it is necessary to establish a food logistics framework from suppliers to retailers based on radio frequency identification technology and design a food logistics tracking system to detect suspicious food to prevent the spread of food safety emergencies (43).

In food supply chain management, the traceability system can measure the efficiency of supply chain operations, reduce information asymmetry, and solve food safety issues and potential food safety incidents (44). Food traceability includes logistics, information, production, and quality management. It is implemented in the food chain based on radio frequency identification and sensor technology to monitor agricultural food safety in real-time (45); this improves the food supply chain management and brings a competitive advantage to the food industry (46). Blockchain is a food traceability method; it establishes a shared safe information exchange record, provides visual and reliable data for transactions (47), which meet the traceability needs of new information from any stakeholder or supply chain node (48), further ensures the sufficient safety of products in the food supply chain, and improves the integrity, reliability, and safety of the food supply chain (49).

#### Cluster 3 (Red): Risk Management of Food Safety in the Food Supply Chain

Food safety issues may appear in all aspects of food production, processing, transportation, and sales. Countries worldwide need to assess and predict food safety risks accurately, confirm the risks, sources, and risk levels of the food supply chain, and maintain transparency and integrity throughout the food industry. The significant food supply chain risks are roughly divided into nine categories: human resource, processing, logistics, raw material, safety certification, traceability, market, packaging, and product characteristic risks. These are related to food manufacturers, transporters, wholesalers, and retailers (13). Researchers use fuzzy analysis of the hierarchical structure process to determine priority food safety risk elements and find that supply related risks are the most prominent ones (50).

The Codex Alimentarius Commission's recommendations to conduct food supply chain management under the acceptable hygiene practices, HACCP systems, and new risk management indicators (such as food safety targets) are indispensable for the optimisation of the food supply chain (51). Food supply chain risks are transmitted through the chain, leading to food recalls and rising costs, and deepening the vulnerability of the global supply chain, especially in terms of food contamination incidents (52). Globalization and the growth of international trade have led to the integration of food safety issues in each country. Countries must formulate consistent food safety standards and measures to coordinate and manage cross-border food trade and use quality management tools to promote global food supply chain risk management systems (53). Simultaneously, mobile technology is essential for food supply chain management, as it helps in improving the food supply chain agility, efficiency, and risk management as well as the economic status of the community, while it reduces risks in the supply chain (54).

#### Cluster 4 (Blue): Food Safety at the Production End of the Food Supply Chain

The COVID-19 pandemic has led to the realization of the importance of food safety and put the focus on the production of agricultural products and heavy metal pollution in the soil (55). Farmers need to monitor the soil to determine the level of metal pollution, establish and maintain the role of soil in the value of the food chain, optimize production systems, and promote a sustainable circular economy (56). All regions should coordinate agricultural production, avoid regional food surplus or shortage, pay attention to seed quality, ensure food production capacity and sales price stability, rely on the supplier to maximize the value of food, and ensure the integrity of the food supply chain (57).

The genetically modified food industry continues to develop rapidly, and safety issues have always been the focus of controversy (58). The vigorous development of biotechnology provides producers in the food supply chain with more opportunities and attracts more attention to genetically modified foods (59). Experts and scholars have found that the main factors affecting consumers' willingness to buy genetically modified food are potential risks, perceived quality, and related social norm risks (60). Simultaneously, studies have found that many consumers in developing countries are more supportive of genetically modified foods than consumers in developed countries.

### Thematic Evolution Trend Analysis

**Figure 8:**
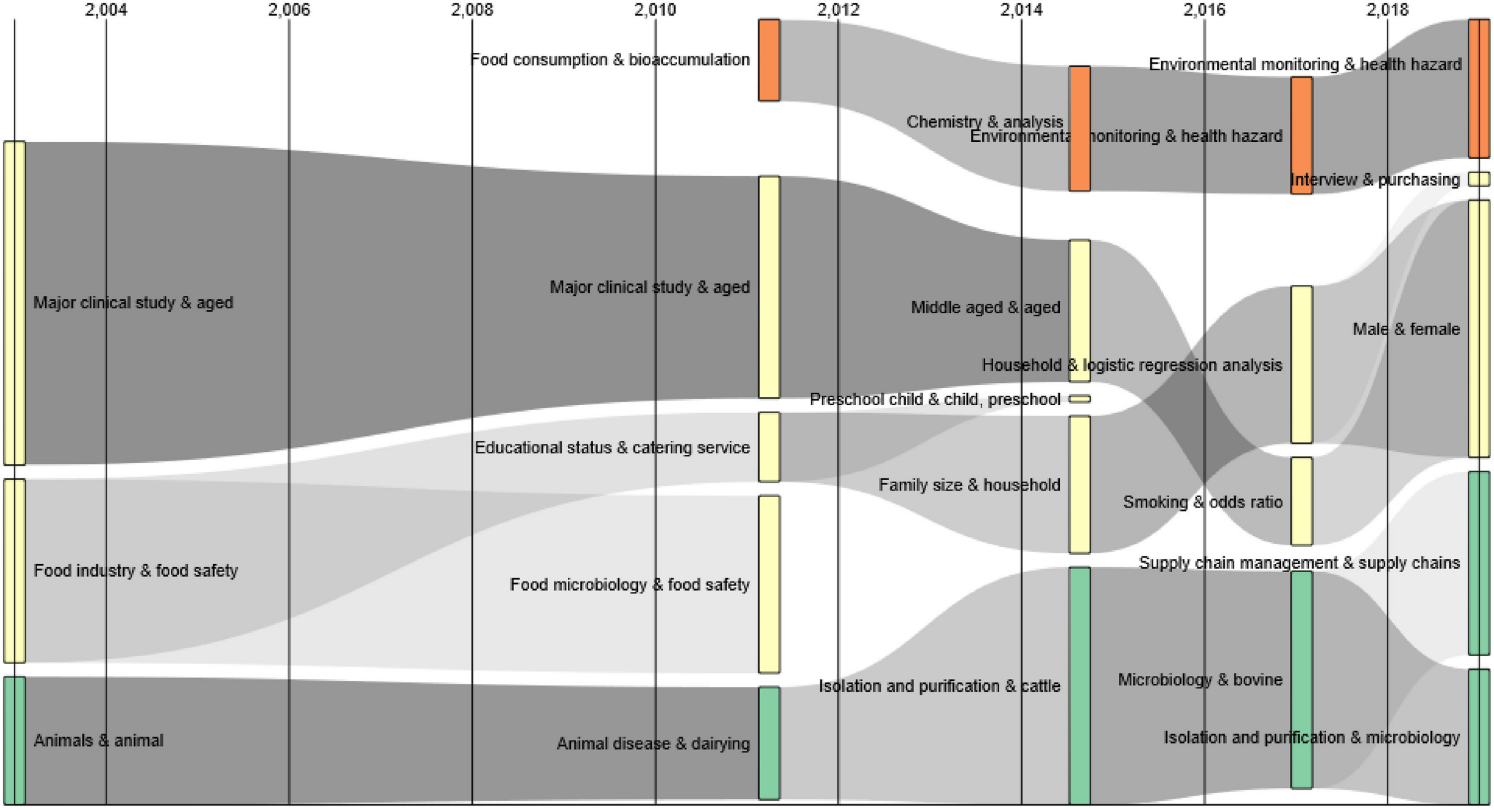
Sankey diagram.

In this study, we analyzed the keywords of 2,329 food safety research articles in the food supply chain field from 1997 to 2020, divided the 24 years into 5-year intervals, and used the CorTexT platform to obtain the evolution of the core topics in the five stages. As shown in the Sankey diagram in Figure 8, the trajectory reveals the evolution of certain food safety research topic characteristics in the food supply chain field over time through the direction and evolution of each time interval's core research topics (61).

Overall, since 1997, food safety research topics in the food supply chain have evolved in terms of emergence, expansion, contraction, differentiation, integration, and extinction in the horizontal time interval; their evolution became more evident after 2012. In 2004, food safety research in the food supply chain field formed three research directions, namely “major clinical study & aged”, “food industry & food safety”, and “animals & animal”. Among these three themes, the one studied the most and the most consistently is the former. Scholars pay more attention to the impact of food safety on consumers of different ages in the food supply chain and clinical research is focused on foodborne diseases (62). The theme of “food industry & food safety” has undergone complex evolution. In 2012, it was divided into “educational status & catering service” and “food microbiology & food safety”. This demonstrates that the food supply chain gradually intersects with various disciplines, such as food safety and consumer safety in combination with research on food microorganisms (63). The theme “animals & animal” formed a continuous evolutionary trajectory. It evolved into “animal disease & dairying” in 2012, and its scale also began to surge. It grew into “isolation and purification & cattle” in 2015 and into “microbiology & bovine” in 2018. It gradually started to differentiate in 2020 and will continue to differentiate into “isolation and purification & cattle” and “supply chain management & supply chains”. This shows that researchers have gradually mastered all aspects of food supply chain management and the overall food supply chain safety management (64), which, combined with the internet and blockchain technologies (65, 66), will help to reduce the food safety incidents.

## Discussion

In this study, we used bibliometric analysis and thematic evolution trend analysis methods to systematically measure and describe the academic research on food safety in the food supply chain and help readers to understand the characteristics of articles published in this field. The number of publications and citations on this topic has been increasing, consistent with the emergence of food contamination incidents in recent years. In particular, the COVID-19 pandemic has swept the world and exposed the fragility of the food supply chain. This situation will attract more scholars to pay attention to food safety incidents in the food supply chain.

Research on the subject classification of articles related to food safety in the food supply chain shows that this field is not limited to categories such as agriculture, food safety, management, and medical care, but is also involved in environmental, social science, and other areas, reflecting the interdisciplinary development of this topic. In the future, scholars need to continue to strengthen multidisciplinary research, improve the level of food safety research, and solve problems in the food supply chain. An increasing number of journals publish articles in this field, showing that academia is very interested in researching food safety in the food supply chain. Additionally, almost all journals examined have high academic standards, and those that impose stricter requirements on authors also have a significant influence on the literary world.

Among the 2,329 examined articles, the first authors' institutions are located in a wide range of countries, covering ~5/6 of the world. Still, most of the authors are from the United States, China, and the United Kingdom. Research countries include developed countries, such as the United States; however, emerging markets such as China are also essential participants in this field. The organizations with the highest number of publications are Wageningen University & Research, the Chinese Academy of Sciences, Harvard University, and the Swedish University of Agricultural Sciences. The authors with the most published research articles are La Vecchia, Talamini, and Jacxsens, and most publications were co-authored by five authors. Researchers from these countries and institutions have done more in-depth and critical research in this field. The analysis and presentation of data pertaining to governments, institutions, and authors will help countries, institutions, and authors cooperate with others, share information and knowledge related to the food supply chain, and use innovative and efficient methods to solve food safety issues.

The top 10 most cited articles listed in the present article are powerful in terms of their arguments and persuasiveness. For example, Roth et al. explain the difficulties and risks inherent in the global food supply chain as a whole and propose six quality management frameworks (traceability, transparency, testability, time, trust, and training) (28). Of course, the number of times an article is cited is also related to the year that the article was published. Therefore, in this study, we used the average number of times, a report was cited in a given year to express more accurately the impact of a publication and provide readers with some context.

In this study, we clustered the keywords that appeared more than 20 times and classified the current four themes of food safety research in the food supply chain based on a visual network map. These themes are food safety at the consumer end of the food supply chain, food safety management in the food supply chain, risk management of food safety in the food supply chain, and food safety at the production end of the food supply chain. The research content mainly focused on meat, the risk safety of genetically modified food in the supply chain (67), and strengthening risk management. Simultaneously, the research focused on analyzing the influencing factors that affect food consumption and explained the importance of safe consumption through quantitative analysis (68) or clinical research. Food safety management protocols should be implemented on all food supply chain stages (69). For specific food contamination incidents, traceability management, especially in the past 2 years, and the emerging blockchain technology have helped improve food supply chain management (70). Different governments educate stakeholders in various links of the food supply chain such as agriculture, consumer markets, cities, rural areas, and households on food safety issues and formulate the relevant policies to enhance food quality and safety for consumers (71, 72). At the same time, governments pay attention to natural factors such as climate change, to achieve the sustainable development of food safety engineering.

By analyzing further, the evolutionary path of keywords and combining this analysis with the clustering results achieved herein, we can conclude that the research hotspots and frontiers of the food supply chain are mainly concentrated on the consumer side of the food supply chain, supply chain management, and the impact of natural factors such as climate on food safety. The four primary directions are entangled with each other in the evolutionary process. The splitting, merging, and reorganization of themes are more pronounced, indicating that the articles are closely related and the degree of differentiation is not high, which suggests that research on food safety in the supply chain still has excellent potential to develop. In particular, blockchain technology in the food supply chain is a research direction that scholars are actively exploring. This interdisciplinary research exemplifies that tackling food safety problems is a systematic task involving agriculture, hygiene, environmental protection, etc., and requires the active participation of all societal sectors.

## Conclusions

In this study, we combined bibliometrics, and thematic evolution trend analysis methods to assess the prominent subjects, journals, research areas, research institutions, and authors in food safety research in the field of the food supply chain. Based on the co-occurrence analysis of keywords, we obtained four main research themes. Next, we performed content analysis and finally analyzed the evolution path diagram. In summary, we explored and examined the thematic evolution, hotspots, and frontiers of research on food safety in the food supply chain. Our results may set the foundations for future research on targeted topics of interest, thereby building a subject knowledge system.

The main contribution of this study was in expanding knowledge in the food safety field. Additionally, we revealed four major research themes and evolutionary paths, highlighted mature and emerging research directions, and proposed new insights into food supply chain safety.

One limitation of this study pertained to data collection from literature databases and the analyzed literature. Specifically, the scope of this study was limited to peer-reviewed publications collected from Scopus. Adding data collected from other databases would expand the publication search. Additionally, in this study, two people were tasked with screening documents as a way of ensuring objectivity. However, a certain degree of subjectivity is bound to remain, especially when selecting the most relevant articles for the final re-analysis.

## Data Availability Statement

The datasets presented in this study can be found in online repositories. The names of the repository/repositories and accession number(s) can be found in the article/Supplementary Material.

## Author Contributions

JL: conceptualization and methodology. SL: data curation and writing—original draft. YB: writing—review & editing and supervision. All authors contributed to the article and approved the submitted version.

## Conflict of Interest

The authors declare that the research was conducted in the absence of any commercial or financial relationships that could be construed as a potential conflict of interest.

## Publisher's Note

All claims expressed in this article are solely those of the authors and do not necessarily represent those of their affiliated organizations, or those of the publisher, the editors and the reviewers. Any product that may be evaluated in this article, or claim that may be made by its manufacturer, is not guaranteed or endorsed by the publisher.
